# Intriguing Role of Proline in Redox Potential Conferring High Temperature Stress Tolerance

**DOI:** 10.3389/fpls.2022.867531

**Published:** 2022-06-10

**Authors:** P. B. Kavi Kishor, Prashanth Suravajhala, P. Rathnagiri, Nese Sreenivasulu

**Affiliations:** ^1^Department of Biotechnology, Vignan’s Foundation for Science, Technology & Research (Deemed to Be University), Guntur, India; ^2^Amrita School of Biotechnology, Amrita Vishwa Vidyapeetham University, Kerala, India; ^3^Consumer-Driven Grain Quality and Nutrition Research Unit, International Rice Research Institute, Los Banos, Philippines

**Keywords:** heat stress, proline cycle, radical scavenging, reactive oxygen species, redox couple

## Abstract

Proline is a proteinogenic amino acid synthesized from glutamate and ornithine. Pyrroline-5-carboxylate synthetase and pyrroline-5-carboxylate reductase are the two key enzymes involved in proline synthesis from glutamate. On the other hand, ornithine-δ-aminotransferase converts ornithine to pyrroline 5-carboxylate (P5C), an intermediate in the synthesis of proline as well as glutamate. Both proline dehydrogenase and P5C dehydrogenase convert proline back to glutamate. Proline accumulation is widespread in response to environmental challenges such as high temperatures, and it is known to defend plants against unpropitious situations promoting plant growth and flowering. While proline accumulation is positively correlated with heat stress tolerance in some crops, it has detrimental consequences in others. Although it has been established that proline is a key osmolyte, its exact physiological function during heat stress and plant ontogeny remains unknown. Emerging evidence pointed out its role as an overriding molecule in alleviating high temperature stress (HTS) by quenching singlet oxygen and superoxide radicals. Proline cycle acts as a shuttle and the redox couple (NAD^+^/NADH, NADP^+^/NADPH) appears to be highly crucial for energy transfer among different cellular compartments during plant development, exposure to HTS conditions and also during the recovery of stress. In this review, the progress made in recent years regarding its involvement in heat stress tolerance is highlighted.

## Introduction

Climate change is on the horizon, and its implications will have a significant footprint on crop productivity and consequently food and nutritional security. Crop plants’ physiological and metabolic activities are radically altered not just by rising day temperatures, but also due to elevated night temperatures. All of the important food crops, such as grains and legumes are prone to high temperature stress (HTS) resulting in an overall reduction in yields (Lobell and Field, 2007; Battisti and Naylor, 2009; Lobell et al., 2011; Xu et al., 2020). HTS negatively affects spikelet fertility, panicle and grain numbers, grain filling, seed size, and grain quality (Peng et al., 2004; Bahuguna et al., 2017; Cheabu et al., 2018; Impa et al., 2019; Dawood et al., 2020). In addition to the yield formation factors altered by HTS, various physiological changes such as decreased photosynthetic efficiency, and formation of reactive oxygen species (ROS) leading to oxidative stress particularly in the chloroplasts occurs with a consequence of membrane damage, and onset of leaf senescence (Fatma et al., 2021). To generate climate resilient crops, it is necessary to understand the underlying physiological and biochemical mechanisms associated with HTS.

**FIGURE 1:**
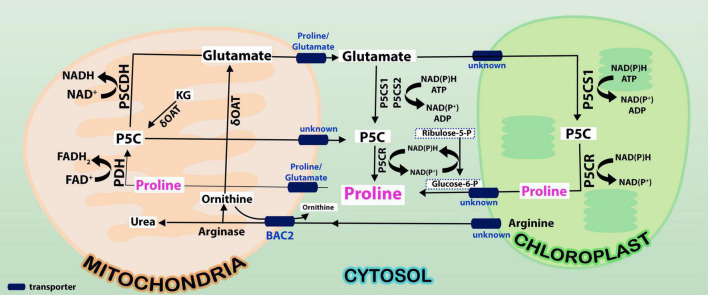
Proline is synthesized from glutamate in cytosol by P5CS1/P5CS2 enzymes and these reactions take place in cytosol (indicated in yellow shade). Oxidation of proline takes place by ProDH and P5CDH enzymes in mitochondria (indicated in green shade). While proline synthesis consumes NAD(P)H and ATP in cytosol, its degradation releases FADH_2_ and NADH in mitochondria. It is not clearly known what transports arginine and ornithine from cytoplasm into mitochondria. But, it is predicted that arginine and ornithine, the two polyamines are transported into mitochondria from cytosol and back by basic amino acid carriers (BAC2). While arginine gets converted to ornithine, ornithine is catalyzed by ornithine δ-amino transferase (OAT) to pyrroline-5-carboxylate (P5C), an overriding intermediate in proline cycle. Note that the release of NAD(P)H is connected to pentose phosphate pathway (PPP pathway) in mitochondria. Conversion of P5C to proline and proline to P5C changes the NADP^+^/NADPH ratio in the cells. This leads to the modulation of phosphoribosyl pyrophosphate (PRPP) synthesis besides nucleotide biosynthesis *via* the PPP pathway.

Touted as a multifunctional amino acid, proline plays vital functions in plant abiotic stress tolerance, including elevated temperature stress (Kishor et al., 1995, 2005, 2015; de Ronde et al., 2000; Kishor and Sreenivasulu, 2014; Mattioli et al., 2020; Maria et al., 2021). *De novo* accumulation of proline was noticed in barley, radish (Chu et al., 1974), leaves of tomato (Rivero et al., 2004), tobacco (Cvikrova et al., 2012), and heat-tolerant varieties of lettuce and wheat in response to heat stress (Han et al., 2013; Djukic et al., 2021). Accumulation of proline has also been found under heat stress in wide array of taxa (Szabados and Savouré, 2010; Pospisilova et al., 2011; Kaur and Asthir, 2015; Harsh et al., 2016; Per et al., 2017). Pyrroline-5-carboxylate synthetase (P5CS) and pyrroline-5-carboxylate reductase (P5CR) are the first two enzymes involved in proline biosynthesis catalyzing glutamate to proline *via* pyrroline-5-carboxylate (P5C; Figure 1). *P5CS* overexpression in transgenic sugarcane resulted in proline buildup under water deficit conditions, which protected chlorophyll and photosystem II (PSII; Molinari et al., 2007). Ahammed et al. (2020) demonstrated that transcription factor *SlWRKY81* represses *SlP5CS1* transcription and drought stress tolerance in tomatoes. On the other hand, *SlWRKY81*-silenced transgenics showed increased proline biosynthesis and drought stress tolerance.

Proline in transgenics has been found to act as a component of antioxidative defense systems, and not for osmotic adjustment (Molinari et al., 2007). Congruently, P5CR in soybean increased the tolerance to heat in Arabidopsis *thaliana* (de Ronde et al., 2004). Existing evidence points out that proline might play a role during osmotic adjustment under heat stress as well as in scavenging the ROS (Kishor et al., 1995; Szabados and Savouré, 2010; Anjaneyulu et al., 2014; Reddy et al., 2015; Signorelli et al., 2015). Experiments carried out by Signorelli et al. (2016) unequivocally demonstrate that proline is not a direct scavenger of peroxynitrite, superoxide, nitric oxide (NO), and nitrogen dioxide (NO2). Contrarily, Hua et al. (2001) and Rizhsky et al. (2004) found no proline accumulation under HTS. Likewise, proline content has been reported to decrease in wheat seedlings during heat stress (Song et al., 2005), inferring that proline accumulation under heat stress is not ubiquitous. In support of this hypothesis, HTS does not trigger the accumulation of proline in *A. thaliana*, and its synthesis literally showed inimical effect (Lv et al., 2011). In contrast, exogenously applied 1 to 5 μM proline protected the Arabidopsis plants exposed to 50^°^C for 10 min (Chua et al., 2020). Foliar application of 5 and 10 mM proline helped in heat stress tolerance in *Capsicum frutescens* exposed to 40^°^C/32^°^C day/night for 30-days and also recovery (Akram et al., 2021). Further, 30 mM foliar application of proline resulted in the alleviation of heat stress in rice seedlings exposed to 34 to 36^°^C (Table 1). Improved antioxidant defense is the hallmark in all these species implying that proline alleviates heat stress through antioxidant defense (Hanif et al., 2021). A list of plants exposed to heat stress and their alleviation by proline and antioxidant defense is shown in Table 1. However, the amount of proline accumulated and the ability to combat heat stress is affected by the nitrogen availability (Rivero et al., 2004). With these contradictory reports, the feasibility of designing crop plants targeting proline biosynthetic pathway genes for heat stress tolerance is still being debated (Verslues and Sharma, 2010; Bhaskara et al., 2015). This difference in proline buildup could be attributed to (a) overexpression of genes involved in proline biosynthesis not only from glutamate but also from ornithine, and (b) proline homeostasis achieved through activation of the proline catabolic pathways. HTS suppressed the expression of proline dehydrogenase (*OsProDH*) in rice (Guo et al., 2020). It appears that proline accumulation quenches the ROS, thereby imparting thermal tolerance. Impaired insulin/IGF1 signaling extends lifespan in worms by boosting proline degradation to induce a transient ROS signal (Zarse et al., 2012), tumor suppression and cell survival (Liu et al., 2006; Phang, 2019), and hypersensitive response in plants (Miller et al., 2009). ProDH generates ROS for signaling, but the threshold levels of catabolic activities that switch survival pathways to cellular apoptosis remain unknown and appear to be an emerging issue. Besides ROS, other players associated with proline metabolic signaling look ambiguous, and a matter in question. To understand the role of biosynthetic and catabolic pathway genes/proteins during heat stress, it is imperative to have a comprehensive idea about their regulatory networks. In this review article, we discuss the role of proline biosynthetic and catabolic pathways to heat response and how the conversion of glutamate to proline and proline to glutamate shuttles the reducing power. Further, we emphasize the importance of proline in radical scavenging and redox potential thereby conferring heat stress tolerance.

## Proline Cycle Modulation by Redox Status, and the Expression of Genes Under High Temperature Stimuli

The regulatory roles of proline and proline cycle in heat stress have been emphasized by Iqbal et al. (2019), Zheng et al. (2021), and Lehr et al. (2022). Through diverse molecular methods, key genes involved in proline biosynthesis and degradation (proline cycle; Figure 1) have been identified in plants (Deuschle et al., 2001). The enzymes P5CS1 and P5CS2 (EC 2.7.2.11, two isoforms) utilize either NADH or NADPH (Chen et al., 2006; Forlani et al., 2017) and ATP to produce P5C and their activities are subjected to feedback inhibition by proline, other amino acids and cofactors (Sabbioni et al., 2021). Millimolar levels of proline have been found inhibitory to rice P5CS2 enzyme. Similarly, analogs of proline like azetidine-2-carboxylate, pipecolate, hydroxyproline, phosphonoproline displayed the ability to bind to the enzyme rice P5CS2 and inhibit the activity aside cofactors such as NAD^+^ and ADP (Sabbioni et al., 2021). These results infer that structural analogs of proline inhibit the P5CS2 enzyme like that of proline feed-back inhibition. Such data on other enzymes like P5CS1, P5CR, ProDH1, ProDH2, and P5C dehydrogenase (P5CDH) are lacking, but the data are critical for better understanding. The findings of Sabbioni et al. (2021) substantiate the influence of redox status of the cell and availability of nitrogen/cofactors like NAD^+^ on proline production.

**TABLE 1:** Correlation between proline and heat stress tolerance in diverse species.

Name of the plant	Temperature applied	Increase or decrease or exogenous supply of proline	Activation of proline pathway enzymes/genes if any	Plant response	References
*Brassica oleracea* var. *capitata*	25 to 35^°^C	Increased proline only up to 35^°^C, not beyond	ND	Heat stress tolerance	Hossain et al., 1995
*Lycopersicon esculentum*	35^°^C	Increased proline	Activation of P5CS enzyme	Heat stress tolerance due to nitrogen source and proline	Rivero et al., 2004
*Triticum aestivum*	25 to 30^°^C for 5-days. For cross-tolerance, seeds pretreated at 33^°^C for various time lengths were transferred to –0.8 MPa NaCl at 20^°^C	Increased proline	Increased P5CS and OAT activities	Heat stress tolerance	Song et al., 2005
*Gossypium hirsutum*	38 and 45^°^C	Decreased proline at 45^°^C	ND	Heat tolerance due to increased antioxidative enzymes	Gur et al., 2010
*Saccharum* sp. cv. HSF-240	42^°^C	Bud soaking in 20 mM proline solution	ND	Heat tolerance and improved bud sprouting	Rasheed et al., 2011
*Triticum aestivum*	25 to 35^°^C	Increased proline	ND	Heat tolerance	Ahmed and Hasan, 2011
*Cicer arietinum*	30/25, 35/30, 40/35, and 45/40°C day/night	10 μM proline	ND (but increased antioxidative defense)	Elevated heat tolerance	Kaushal et al., 2011
*Nicotiana tabacum* (transgenic)	40^°^C for 1 h	Increased proline to heat stress/drought	Overexpression of *P5CSF129A* mutated gene from *Vigna aconitifolia*	Correlation between heat stress and increased proline was not proved	Pospisilova et al., 2011
*Arabidopsis thaliana* (transgenic)	37^°^C for 24 h first and then to 50^°^C for 4 h	No accumulation of proline under heat stress	Overexpression of *AtP5CS1*	Proline increases ROS, inhibits ABA and ethylene synthesis, and decreases thermotolerance (ABA and ethylene rescue heat-sensitive phenotype)	Lv et al., 2011
*Nicotiana tabacum* (transgenics)	40^°^C for 2 hs	Increased proline in wild type plants after 2 h, but after 6 h in transgenics	Overexpression of *P5CSF129A* mutated gene from *Vigna aconitifolia*	Heat stress tolerance due to proline and polyamines	Cvikrova et al., 2012
*Sorghum bicolor*	40^°^C for 6 h	Increased proline	Increased P5CS activity	Heat stress tolerance	Gosavi et al., 2014
*Solanum lycopersicon*	35^°^C heat stress for 48 h Combined heat and salinity stress	Decreased proline Increased proline	Decreased P5CS, P5CR, Proline oxidase and P5CDH Decreased P5CS, P5CR, but increased OAT	- Heat tolerance due to high K^+^ and lowered Na^+^/K^+^ ratio	Rivero et al., 2014
*Prunus persica*	Two heat treatments ∼23.5/19.2°C day/night and one cold treatment for 14-days Two heat treatments over 4 days of 24.1/18.7°C Day/night for 4-days and then cold treatment	Proline accumulated No change in proline levels	OAT upregulated, P5CR downregulated OAT, P5CR were downregulated	Proline accumulation increased associated with the preparation for growth resumption Ornithine pathway appears important for proline synthesis	Shin et al., 2016
*Vigna aconitifolia*	42^°^C	Increased proline	ND	No heat tolerance	Harsh et al., 2016
*Triticum aestivum*	38^°^C for 4 h	Increased proline in the initial stages, and then decrease at later stages	ND (but increased antioxidant defense)	Heat stress tolerance	Mishra et al., 2017
*Festuca trachyphylla*	38/33^°^C day/night	Increased proline	ND	Heat tolerance	Wang et al., 2018
*Pisum sativum*	44^°^C for 4 to 8 min (hyper thermal stress)	Increased proline	ND	Heat tolerance	Nesterenko and Rashydov, 2018
*Glycine max*	40°C for 14 h and 30°C for 10 h for 5-10-days	Increased proline with inoculation of *Bacillus cereus* isolate SA1	ND	Significant heat tolerance	Khan et al., 2020
*Triticum aestivum*	Osmotic potential -1.47 MPa	Increased proline	ND (but increased antioxidan t defense)	Combined heat and drought stress alleviation	Sattar et al., 2020
*Oryza sativa*	45^°^C for 48 h	High temperature repressed the expression of *OsProDH* and increased proline	*OsProDH* overexpression and knockout lines	Overexpressed lines were sensitive to heat, but knockout lines produce higher proline content and tolerant to heat stress	Guo et al., 2020
*Arabidopsis thaliana*	25, 35, 45, 50, 55, 65, 75, or 85^°^C, and heat stressed for 10 min. 50^°^C for 10 min	Uptake of cyanobacteria-derived- proline alters root hair programmed cell death (PCD) sensitivity threshold. Exogenously applied 1 to 5 μM proline	ND	Heat stress tolerance with reduced PCD Heat stress tolerance	Chua et al., 2020
*Solanum lycopersicum*	Heat and salinity applied in combination	Increased proline	Proline and ascorbate pathways act synchronously to maintain cellular redox homeostasis	Increased heat stress tolerance	Lopez-Delacalle et al., 2021
*Triticum aestivum*	34 to 35^°^C	Increased proline	ND	Mitigation of heat stress	Djukic et al., 2021
*Oryza sativa*	34 to 36^°^C	Foliar application of 30 mM proline	ND (increased antioxidant defense)	Alleviation of heat stress	Hanif et al., 2021
*Capsicum frutescens*	40/32^°^C day/night for 30-days	Foliar application of 5 mM and 10 mM proline	ND	Heat stress tolerance and recovery	Akram et al., 2021
*Capsicum frutescens*	42^°^C for 10 days	Increased proline	ND	Faster recovery from heat damage	Rajametov et al., 2021
*Vitis vinifera*	39 and 25^°^C (maximum and minimum)	Increased proline	High expression of *P5CS* gene	Heat, combined heat and drought stress tolerance	Lehr et al., 2022

*ND, Not determined.*

### Localization of P5CS1, P5CS2, Pyrroline-5-Carboxylate Reductase, Proline Dehydrogenase, and Pyrroline-5-Carboxylate Dehydrogenase Proteins and Redox Modulation

While P5CS1 is required for the synthesis and accumulation of proline under stress treatments, P5CS2 is indispensable for embryo and seedling development (Funck et al., 2020). Evidence has also been presented that both P5CS1 and P5CS2 participate in the synthesis of proline (Funck et al., 2020). Initially, it was predicted that P5CS1 protein is localized in plastids and P5CS2 in cytoplasm (Szekely et al., 2008). Subsequent experiments with fluorescence imaging technique showed that both P5CS1 and P5CS2 are located in cytosol in Arabidopsis, and plastids do not contribute to the synthesis of proline (Funck et al., 2020). Thus, subcellular localization of P5CS1 is still enigmatic in plants. Possibly, its localization may depend upon the growth or stress conditions that the plants are undergoing. In line with this, *p5cs1* mutants of *A. thaliana* accumulate ROS due to upregulation of genes implicated in ROS but not the overexpressed lines (Szekely et al., 2008; Shinde et al., 2016). It has been shown that alternative splicing of AtP5CS1 results in natural variation in the content of proline and climate adaptation (Kesari et al., 2012) implaying the importance of P5CS during stress. With regard to redox under abiotic stress conditions, Shinde et al. (2016) have noticed an interconnection between proline and lipid metabolism which may help in buffering cellular redox status. On the other hand, P5CR (EC 1.5.1.2, four isoforms in all including bacterial sources) uses either NADH or NADPH as the electron donor with contrasting affinities and maximum reaction rates (Forlani et al., 2017). P5CR is mostly localized in cytosol in *A. thaliana* (Funck et al., 2012). Activity of P5CS was inhibited by cations like Na^+^, Mg^2+^, and Ca^2+^, anions such as Cl^–^ promoted it (Sabbioni et al., 2021). Interestingly, in *A. thaliana*, stimulation or inhibition by chloride ions and feedback regulation by proline depends on whether NADPH or NADH acts as co-substrate (Giberti et al., 2014). This infers that activities of P5CS and P5CR lower the concentrations of NADPH/NADP^+^ ratio in the cytosol (Liang et al., 2013; Zheng et al., 2021, Lehr et al., 2022). While proline suppressed the activity of only NADH-dependent enzymes, salt stimulated the NADPH-dependent reaction (Giberti et al., 2014). Further, the catabolic pathway enzymes like ProDH (EC 1.5.5.2) and pyrroline-5-carboxylate dehydrogenase (P5CDH, EC 1.2.1.88) are also dependent on FAD and NAD(P) for their activities, respectively. While ProDH is localized on the matrix side of the mitochondrial inner membrane (Cabassa-Hourton et al., 2016), P5CDH (single copy in Arabidopsis) has been found in the mitochondrial inner membrane in maize.

### Generation of Reactive Oxygen Species by Pyrroline-5-Carboxylate and Proline Cycle

**FIGURE 2:**
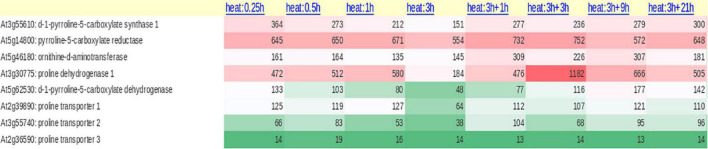
Expression of proline biosynthetic pathway genes (anabolic and catabolic) both from glutamate and ornithine and proline transporters (ProT) under diverse temperature regimes in *Arabidopsis thaliana*.

The enzymes ProDH and P5CDH were found to interact with drought and freezing responsive gene 1 (DFR1) protein physically. DFR1 protein mediates the inhibition of proline degradation and modulates drought and freezing tolerance (Ren et al., 2018). P5CDH is also crucial to degrade the toxic effects of proline in plants or its intermediate glutamic semialdehyde/P5C to glutamate releasing the reducing power in mitochondria. In plants, proline-P5C cycle generates ROS which can trigger cell apoptosis and prevent pathogenesis. Such proline/P5C toxicity could be because of an overflow of electrons in the mitochondrial electron transport chain (mETC). The increase in ROS was caused by inhibiting P5CDH or yeast mutants lacking this enzyme, demonstrating that P5C functions as a stress response regulator and that its levels must be strictly maintained (Deuschle et al., 2001; Zheng et al., 2021). Thus, the redox state, as well as chloride and proline concentrations in the cytosol, govern all enzymatic activities of the proline cycle in a complicated way. Since proline metabolism is linked to cellular compartments like mitochondria and energetics, it takes part in NAD(P)^+^/NAD(P)H homeostasis during the growth of plants and when exposed to the stress conditions or relieved from it. A look at the expression of the genes involved in proline cycle and the transporters (eight identified in Arabidopsis) infers that the genes are up- and down-regulated by HTS (Figure 2). Surprisingly, more than *P5CS*, *P5CR* gene was upregulated under HTS indicating its importance during short- and long-term exposure to temperature stress. *Ornithine*δ*-aminotransferase* (*OAT*) also was expressed but under long-term exposure, while its expression was not altered under short-term treatments.

### Proline Oxidation Maintains NADP/NADPH Ratio in Cells

Once the stress is relieved, proline is transported to mitochondria either through proline symporter or a proline/glutamate antiporter to produce energy [FADH_2_ and NAD(P)H] by ProDH, and P5CDH, respectively, and release glutamate (Di Martino et al., 2006). But the genes that encode these transporters have not yet been identified in plants. It appears now that glutamate released into mitochondria is transported back to cytosol *via* a novel glutamate transporter “a bout de souffle” (BOU) and uncoupling proteins1 and 2 which also act as transporters of aspartate and dicarboxylates (Monne et al., 2018; Porcelli et al., 2018). Shuttling of proline and glutamate and also the reducing power between the cellular compartments like cytosol and mitochondria is vital for maintaining proper ratio of NAD(P): NAD(P)H in the cellular system. In contrast, *ProDH* was consistent in its expression especially at 3 + 3-h-long temperature stress exposure, but not *P5CDH* barring a moderate expression at 3 h/4 h treatments (Figure 2). Though transcript levels of *OsProDH* were high in root and leaf blade, heat stress reduced the transcript levels of *OsProDH* (Guo et al., 2020). Expression of the *ProDH1* and *ProDH2* genes has been found upregulated during plant senescence (Launay et al., 2019). This may help to reduce the toxic levels of proline/P5C on one hand and on the other, aid to generate energy (Launay et al., 2019). These results distinctly demonstrate that proline oxidation fuels mitochondrial respiration during senescence which is essential for maintaining NADP^+^ and NADPH ratio in the cells. Proline cycle is therefore indispensable in the cells for maintaining proper cellular redox. Taking cognizance of these studies, breeding strategies must be developed to enhance HTS tolerance by manipulating proline metabolism.

### Long Distance Transport of Proline Is Vital for Stress Tolerance

Though proline biosynthesis occurs in cytosol and chloroplasts (Szabados and Savouré, 2010), proline is detected both in xylem and phloem (Lehmann et al., 2010), inferring its transport in different tissues. While Girousse et al. (1996) noticed water stress-stimulated transport of proline to long distances *via* phloem in alfalfa, Mattioli et al. (2020) demonstrated its accumulation in pollen under various abiotic stress conditions, so, its transport to pollen grains is essential for the pollen fertility, thereby limiting the seed loss. Proline porters also act as porters of betaine and γ-aminobutyric acid in *A. thaliana* (Breitkreuz et al., 1999). In *Arabidopsis thaliana*, the expression of proline transporter 3 (*AtProT3*) was steady under both short-term and long-term exposure to temperature stress compared to *AtProT1* and *AtProT2* (Figure 2). However, the functions of other ProTs under heat stress conditions are unclear. These studies clearly point out that along with the proline cycle genes, transporters are also vital for stress tolerance, under normal as well as environmental stimuli. There is also a debate about whether proline buildup is more important to confer short-term stress tolerance or attaining proline homeostasis through the activation of a catabolic pathway to meet the energy and redox potential in driving plant development under prolonged stress (Kishor and Sreenivasulu, 2014).

### Production of Proline From Ornithine Depends on Cellular Status of Proline or Glutamate Levels

**FIGURE 3:**
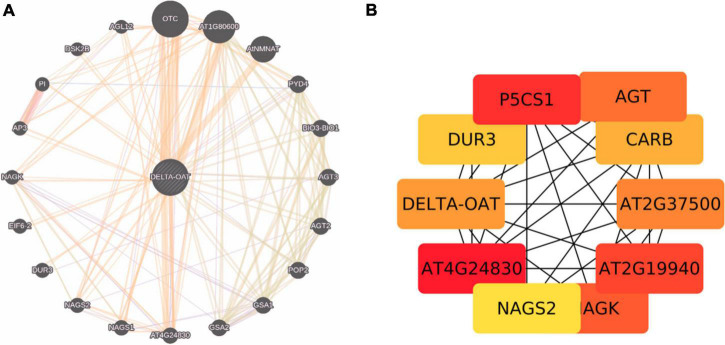
**(A)** Protein interaction networks generated by genemania.org taking OAT and its interacting partners. **(B)** Cytohubba prediction of OAT with ranked genes. Brighter the node, larger is the adherence of accumulated stress protein.

Proline is synthesized not only from glutamate but also from ornithine (derived from arginine) by OAT (Chalecka et al., 2021). Shin et al. (2016) have presented evidence that ornithine, rather than glutamate pathway is the major route for the synthesis of proline both during growth resumption and HTS in *Prunus persica*. Production of proline from ornithine involves P5C, the intermediate in the cycle. But, Funck et al. (2008) and Winter et al. (2015) showed that P5C derived from ornithine is utilized for generation of glutamate *via* P5CDH rather than proline *via* P5CR. This may perhaps depend on cellular status of proline or glutamate levels and also stress conditions. If the levels of P5C are higher, which are toxic to the cells, it may get converted to glutamate. So, P5C is at a central point to deal with the situation depending on cellular conditions (Anwar et al., 2018; Chalecka et al., 2021). It has also been pointed out that proline regulates the function of mitochondria influencing the death of cells when biotrophic and necrotrophic pathogens invade and in cancer state (Rizzi et al., 2016; Phang, 2019). Given the adage that the OAT is an important stress associated protein involved in proline/glutamate biosynthesis, it has been sought to identify the interacting partners of the protein (Figures 3A,B), which are largely co-expressed with a few of the experimentally determined genes (indicated in yellow background in Supplementary Table 1). We explored important nodes/hubs from the interaction using stress as a topological algorithm from Cytohubba app (Chin et al., 2014) of cytoscape. Highly ranked proteins have been identified during mining (Figures 3A,B) from stress using the Edge Percolated Component, Maximum Neighborhood Component, Betweenness and Closeness Centralities, Density of Maximum Neighborhood Component, Maximal Clique Centrality, and centralities based on shortest paths, such as Bottleneck, and radiality. Network hub indicates that P5CS1 is also one of the key interaction partners of OAT under stress. An attempt was made to unravel the connections between OAT protein and lncRNA targets under stress, but apparently none of the bonafide genuine possibilities were uncovered. This systems biology prediction is in agreement with the supported hypothesis that with an increased OAT, there is an alteration in stress-regulated P5CS1 levels.

## Protection of Photosynthetic Machinery and Suppression of Programmed Cell Death Under High Temperature Stress

High temperature stress causes many changes in plant growth, development, leaf senescence, discoloration of leaves (Vollenweider and Gunthardt-Goerg, 2005), degradation of chlorophyll *a* and *b* (Jahan et al., 2021), and decline in quantum efficiency. Functional aspects of chloroplasts during programmed cell death (PCD) are mostly obscure. Zavafer et al. (2020) noticed that chloroplasts integrity is collapsed which leads to PCD promoted by long chain bases or ceramides under HTS. In response to HTS, metabolic reprogramming such as breakdown of chlorophyll, production of ROS and alterations in carbon metabolism occur. Such an alteration in metabolic programming is perhaps essential during stress acclimation. Chloroplasts play a crucial role in inducing the expression of nuclear heat-response genes under HTS response (Hu et al., 2020). Light intensity inhibits chlorophyll synthesis especially if ABA levels are low or poor like in *nced3nced5* mutants (Huang et al., 2019). High light and HTS produce ROS in the thylakoid which play vital roles as signal transducers. ROS generated during light and HTS provide cells with vital information on the current status of abiotic stress (Foyer and Noctor, 2016; Foyer et al., 2017). Heat stress causes reduced leaf nitrogen concentrations, protein damage, oxidative stress, and associated membrane damage, limiting plant growth and productivity (Wahid et al., 2007; Fahad et al., 2017). Temperature stress lowers chlorophyll biosynthesis due to decreased chlorophyll biosynthetic pathway enzymes such as 5-aminolevulinate dehydratase (Dutta et al., 2009), which could be linked to oxidative damage (Guo et al., 2006; Mohanty et al., 2006). Proline is associated with the protection of thylakoid membranes against free radical-induced photodamage (Alia et al., 1997). Interestingly, reduced ratio between chlorophyll to carotenoid was recorded in tomato and sugarcane plants that are heat tolerant (Camejo et al., 2005; Huang et al., 2019) inferring that pigment (chlorophyll to carotenoid) ratio is implicated in temperature stress tolerance. PSII is highly temperature sensitive and its activity is either partially or totally impaired depending upon the duration and intensity of heat stress (Camejo et al., 2005; Huang et al., 2019). High day and night temperatures perturb the photosynthetic activity in rice cultivars (Fahad et al., 2016). *Glycine max* transgenic lines containing *P5CR* gene (sense and antisense) were used to study the effect of heat stress. While sense plants reveal elevated proline alongside high NADP**^+^** levels with no symptoms of temperature stress, antisense plants showed lower levels of NADP**^+^** with severe symptoms (de Ronde et al., 2004). These results corroborate what has been noticed in the heat map (Figure 2). NADP**^+^** concentrations reached normal levels faster in sense plants during rewarming from stress in comparison with antisense plants. Furthermore, after photoinhibition or repeated heating in all plants, the oxygen evolving complex, D1 and D2 proteins dissociate, disrupting electron transfer to the acceptor molecule PSII and reducing RuBP regeneration (Sharkova, 2001; Wise et al., 2004; Toth et al., 2005). Thus electron transport is the functional limitation of photosynthesis at higher temperatures. Plants cope better with heat stress, where proline supplies electrons to PSII (Moustakas et al., 2011; Oukarroum et al., 2012). This type of electron transport in PSII keeps NADPH levels normal and protects the PSII from additional damage (Oukarroum et al., 2012). When plants are relieved from stress, proline is catabolized releasing high concentrations of NADP**^+^** which would be used for accepting the electrons in PSII which may ameliorate the effect of uncoupling of the oxygen evolving complex (de Ronde et al., 2004). Thus, proline plays a key role in protecting the PSII and other events related to photosynthetic machinery and its activity under HTS.

Interestingly, heat-stressed *A. thaliana* plants that were pre-treated with *Nostoc muscorum* displayed lower PCD in root hairs in comparison with untreated seedlings. By suppressing PCD but not necrosis, cyanobacteria (*N. muscorum*)-derived proline enhanced the heat stress tolerance in *A. thaliana* root hairs (Chua et al., 2020). Also, exogenous supply of proline mimicked comparable PCD suppression levels like that of *N. muscorum* (Chua et al., 2020). Further, proline transporter mutants like *lht1*, *aap1*, and *atprot1*-*1:atprot2-3:atprot3-2* were used to find out if proline is playing any active role during heat stress. Suppression of PCD under heat stress in *lht1* and *aap1* mutants was reduced markedly when 5 μM levels of proline were supplied exogenously. When the three mutants were pre-treated with cyanobacteria, PCD levels were comparatively enhanced (Chua et al., 2020). Thus, these experiments clearly reveal that *Nostoc*-derived proline protects the plants by interfering with PCD activity. Nevertheless, such mechanisms need to be studied in depth to understand the implication of proline in preventing PCD of plants under HTS.

## Proline as a Quencher and a Scavenger of Reactive Oxygen Species Generated During High Temperature Stress

Under normal conditions, equilibrium between the production of ROS and antioxidant defense system is maintained, which is perturbed under abiotic stress conditions including HTS. HTS leads to the high accumulation of ROS and oxidative stress in plants. But the production of ROS and their detoxification by both enzymatic and non-enzymatic machinery must be maintained in such adverse environmental conditions (Hasanuzzaman et al., 2020), though our understanding about ROS signaling is limited. Ectopic overexpression of *Stipa* P5CS gene (*StP5CS*) in *A. thaliana* resulted in higher survival rates of plants under drought stress with less membrane damage and superior antioxidant machinery (Yang et al., 2021). HTS elevated the expression of *P5CS* in addition to heat shock protein (*HSP*), and manganese superoxide dismutase 1 (*MSD1*) in rice (Nahar et al., 2022). Their data display that the responses of plants to salt, heat and a combination of salt + heat are unique, but complex with many molecular network modules. Clearly, mitigation of salt and heat stresses were observed with improved antioxidant enzyme activities and elevated expression of *P5CS* gene and subsequent proline accumulation. Foyer et al. (2017) has proposed that oxidative stress must be viewed as a signaling mechanism rather than damage to the cells.

Reactive oxygen species such as hydroxyl radical, hydrogen peroxide, superoxide, and singlet oxygen are produced due to environmental conditions including heat stress as pointed out. ROS can denature proteins, lipids and many macromolecules and inactivate photosynthetic machinery. But, enzymatic and non-enzymatic (ascorbate, a-tocopherol, glutathione, etc.) machinery exists to remove the ROS from the system. Again, the putative role of proline as an antioxidative molecule has sparked debate. Proline is thought to be a ROS or hydroxyl radical quencher (Matysik et al., 2002; Signorelli et al., 2014, 2015). It is known that proline reduces or eliminates the levels of hydroxyl radical and hydrogen peroxide *in vivo* in non-transgenic tobacco and improves the antioxidative enzymatic activities in transgenic *Sorghum bicolor* plants under stress (Banu et al., 2010; Reddy et al., 2015) and also increases glutathione under drought and heat stresses in *G. max* (Kocsy et al., 2005). The initial evidence points out that proline cannot totally quench or scavenge singlet oxygen, and does not interact with superoxide radical, nitric oxide, peroxynitrite, and nitrogen dioxide (Signorelli et al., 2013, 2016; Signorelli, 2016). Rehman et al. (2021) used a variety of techniques, including electron paramagnetic resonance spin trapping with 2,2,6,6-tetramethyl-4-piperidone (TEMPD), fluorescence probing with singlet oxygen sensor green (SOSG), and oxygen uptake in isolated thylakoids, to demonstrate that proline quenches both singlet oxygen and superoxide radical *in vitro via* an electron transfer reaction. Their experiments demonstrate that singlet oxygen-scavenging capacity of proline reaches up to two thirds that of α-tocopherol and significantly superior or the same that of ascorbate (Rehman et al., 2021). Further, Aswani et al. (2019) have shown that proline plays a physiological role in singlet oxygen detoxification in the leaves of *Pisum sativum* exposed to methyl viologen that generates large amounts of singlet oxygen under light conditions. Proline makes physiologically meaningful contributions to ROS (singlet oxygen) quenching, and may operate as a supplement to other non-enzymatic ROS quenchers in stressful settings, according to these investigations.

## Does Proline Play a Vital Role in the Regulation of Redox Potential Conferring Heat Stress Tolerance?

It has been demonstrated that P5C-proline cycles operative in different cellular compartments are associated with signaling events and modulation of intracellular redox potential (Hare and Cress, 1997; Miller et al., 2009). Redox homeostasis is essential during photosynthesis especially under the conditions of climate change to promote plant development (Scheibe et al., 2005). Proline metabolism is compartmentalized mostly in chloroplasts, mitochondria and cytoplasm implying that reducing equivalents are concentrated in these compartments (Lunn, 2006). Regulation of energy in the form of ATP, and reducing power (NADH/NADPH), its homeostasis under heat stress requires proper channelization and utilization wherever necessary (Foyer and Noctor, 2020). Dark CO_2_ fixation is affected under heat stress since stomata are closed and chlorophyll molecules are degraded leading to reduced generation of reducing power in chloroplasts. Since carbohydrate supply is limited under heat stress, concomitant decrease in ATP and NADPH generation is generally noticed in mitochondria. Therefore, very tight spatiotemporal regulation and supply of energy at different cellular compartments is necessary under heat stress. While NAD^+^ is synthesized in the cytoplasm, it must be translocated into chloroplasts and mitochondria *via* NAD^+^ carrier proteins NDT1 and NDT2 localized in plastid and mitochondrial membranes (Palmieri et al., 2009). NDT1, the mitochondrial transporter, appears to play a crucial role in cellular NAD^+^ homeostasis in *A. thaliana* (de Souza Chaves et al., 2019). However, the redox couples (NAD^+^/NADH and NADP^+^/NADPH) produced by calmodulin-dependent NAD^+^ kinase (Gakiere et al., 2018; Dell’Aglio et al., 2019) are not permeable to organellar membranes and must be transported across subcellular compartments by shuttle systems or metabolic valves. Under abiotic stress conditions, the glycerol-3-phosphate, malate-aspartate shuttle for NADH pools, and malate-oxaloacetate shuttle for NADPH pools transport reducing equivalents from subcellular compartments into the cytoplasm and back, maintaining redox and energy homeostasis. As part of proline cycle, P5CS and P5CR convert glutamate to proline using NAD(P)H, as an energy source; while ProDH and P5CDH in the mitochondria accelerate the conversion back to glutamate, lowering both FAD^+^ and NAD^+^ molecules (Liang et al., 2013). This cycle thus involves NAD(P)^+^/NAD(P)H between cytoplasm and mitochondria during plant development and also during abiotic stress including heat stress. Arabidopsis *p5cs1* mutants exhibit sensitivity to salt stress, accumulate ROS with implications in redox metabolism at subcellular compartments like chloroplasts and mitochondria (Szekely et al., 2008; Shinde et al., 2016). Ruszkowski et al. (2015) and Shinde et al. (2016) noticed that proline metabolism helps in maintaining cellular redox under stress conditions. Proline and ascorbate pathways have been found to act synchronously to maintain cellular redox homeostasis in tomato (Lopez-Delacalle et al., 2021; Table 1). Interestingly, it has been found that NADP^+^ suppresses the P5CR expression, but not NADPH-dependent reactions (Giberti et al., 2014). Further, NADH-dependent P5CR activity is reduced if proline is accumulated in excess. Thus, proline cycle helps in the production of FADH_2_ and NADH which might impact the electron transport in mitochondria alongside the production of ROS. Huang et al. (2013) also reported the discovery of succinate dehydrogenase (SDH) assembly factor 2 (SDHAF2), a protein that co-expresses with SDH1-1 and is required for the insertion of FAD^+^ into SDH component or mitochondrial complex II, as well as proper root elongation in *A. thaliana*. Further, it has been shown that proline biosynthesis might act as a redox vent even in mammals (Schworer et al., 2020).

Under stress conditions, proline accumulation assists in plant growth. But application of proline under normal conditions inhibits plant growth (Han et al., 2021). If proline is supplied externally under normal conditions, degradation of excess proline induces mitochondrial ROS due to electron overflow in mETC which might contribute to the proline toxicity and inhibition of plant growth. A mitochondrial truncated matrix protein (SSR1) with a tetratricopeptide repeat domain has been shown by Han et al. (2021) to be involved in maintaining the function of mETC in proline hypersensitive phenotype of *A. thaliana*. When the *ssr1-1* mutant was given proline, accumulation of ROS was dramatically increased due to the increased activity of ProDH, resulting in proline breakdown and triggering of apoptosis (White et al., 2007; Pandhare et al., 2009). Han et al. (2021) pointed out that treating plants with proline under normal conditions might lead to higher mitochondrial ROS, lower ATP content, decreased mETC complex I and II. These results imply that SSR1 is associated in maintaining mETC at optimum levels to mitigate proline toxicity under normal conditions. Because ProDH binds to the coenzyme Q, superoxide radicals may not be formed during proline oxidation by ProDH at complex II, as they are produced at complex III (Goncalves et al., 2014; Hancock et al., 2016). Furthermore, polymorphisms in mitochondrial DNA were linked to two genes that encode NADH dehydrogenase subunits. These findings show that proline accumulation and cellular redox are carefully regulated, with proline breakdown being the most important factor in stress tolerance (Atkin and Macherel, 2009; Sharma et al., 2011). Aside from providing energy in the form of FADH_2_ and NADPH, proline degradation impacts oxidative stress resistance. Zhang et al. (2015) characterized oxidative stress resistance of *putA* (contains both ProDH and P5CDH domains) mutant strains of *E. coli*. These experiments revealed that *putA* mutants are sensitive to oxidative stress compared to the wild-type strain. Thus, proline serves as an important molecule in oxidative stress resistance.

## Why Is Proline Oxidation Crucial During Stress and Stress Release?

Proline degradation is equally essential for supplying energy under long term stress and when the plants are relieved from stress. Proline is synthesized in chloroplasts/cytoplasm, but transported through proline porters to the root and shoot tips where it supplies energy by oxidation in mitochondria. During senescence, degradation of Calvin cycle enzymes occurs, therefore, NADPH levels are reduced in the senescing leaves. The accumulated leaf proline activates ProDH leading to higher activity of ProDH1 and ProDH2 under senescing conditions (Launay et al., 2019; Dellero et al., 2020). Thus, oxidation of proline during heat stress and also during stress release is an important additional source of energy besides replenishment of NADP^+^/NADPH ratio in the cells. Besides ProDH, P5CDH also contributes to the alleviation of oxidative stress. Mutants of *p5cdh* accumulate ROS when proline was supplied exogenously (Deuschle et al., 2004; Miller et al., 2009). The results reveal that *P5CDH* and the dynamics proline cycle are important for maintaining ROS homeostasis.

Thioredoxin (Trx) is a ubiquitous thiol-disulfide reductase and the master regulator protein of the tricarboxylic cycle in plant mitochondria, and located both in mitochondria and cytosol. Trx regulates SDH, fumerase and ATP-citrate lyase by modulating thiol redox status, and thus the carbon flux. The NADPH produced by dehydrogenases including P5CDH can be utilized in glutathione reductase for protection against oxidative stress and by thioredoxin reductase in the regulation of metabolic pathways (Moller and Rasmusson, 1998). In other words, proline catabolism plays a role since Trx makes use of NADPH through NADPH-thioredoxin reductase enzyme (NTR; Geigenberger et al., 2017). In comparison to wild-type plants, *trxo1* mutant and *ntra*, *ntrb* double mutants amass less proline but more glutamate and malate (Daloso et al., 2015; Geigenberger et al., 2017). Malate serves as a source of NADH/NADPH in the TCA cycle under stress conditions. Accumulation of proline was curtailed in the mutant *trx1* under drought stress conditions (Verslues et al., 2014). Trxs not only transmit the light signal from chloroplasts to mitochondria, but also moderate the enzymes of the proline cycle. Importantly, when chloroplasts are inactive during night time, proline degradation may release energy (FADH2 and NADPH) and redox power to the cells. Therefore, proline degradation and the energy released thereof is imperative unequivocally once the plants are relieved from stress. Plants accumulate 56-times more amounts of proline in flowers than the leaves (Schwacke et al., 1999; Kishor and Sreenivasulu, 2014). Proline also accumulates significantly in high concentrations in developing microspores from local synthesis and accounts for 70% of the total free amino acids. Proline has been found essential for pollen fertility and improved yield stability under salt stress in *A. thaliana* (Mattioli et al., 2018, 2020). Further, tomato proline transporter LeProT1 is expressed in the germinating pollen tube and supplies energy during the growth of the pollen tube (Mattioli et al., 2018).

## Cross Talk Between Proline and Polyamine Metabolism During High Temperature Stress

High temperature stress activates the accumulation of proline and polyamines (Chakraborty and Tongden, 2005; Cvikrova et al., 2012). Anabolism and catabolism of polyamines have been noticed in plant leaves exposed to heat stress (Walter, 2003). Proline and polyamines play a role in osmotic adjustment as well as in scavenging ROS (Bouchereau et al., 1999). The effect of heat stress on the accumulation of polyamines and proline in transgenic tobacco plants (overexpressing *P5CSF129A* gene with no feedback regulation of P5CS enzyme) that overproduce proline in lower, and upper leaves, and roots was recorded (Cvikrova et al., 2012). Genes that encode proline biosynthetic pathway enzymes confer salt stress tolerance in *Panicum virgatum* in cooperation with polyamines metabolism (Guan et al., 2020). Proline accumulated after 6-h of heat stress treatment in the lower leaves. After 2-h of exposure to 40^°^C, wild-type tobacco plants accumulated more proline than transgenics, but after a 2-h lag period, transgenics accumulated putrescine, spermidine, norspermidine, and spermine, with matching increases in enzyme activity (Cvikrova et al., 2012). During the initial heat stress, polyamine oxidase was elevated in the roots of wild-type and transgenic plants, but it was unexpectedly reduced in the leaves of transgenics. Decrease in the activity of ornithine decarboxylase and increase in the diamine oxidase in the leaves and roots were recorded in transgenics (Cvikrova et al., 2012). In addition, the results obtained by Cheng et al. (2009) in tomato infer that both spermine and spermidine play indispensable roles in heat stress tolerance. High levels of proline in transgenics also appear to play a positive effect on heat stress tolerance, since degradation products of proline aid in the biosynthesis of polyamines in the early stage of exposure to heat (Cvikrova et al., 2012). Changes in the accumulation of proline and its metabolism have been found regulated depending on the age of the leaf in pea plants exposed to metal stress, independent of ABA signals (Zdunek-Zastocka et al., 2021). Such an accumulation of proline in the leaves may help in osmotic adjustment during stress.

## Role of Carbohydrates, Nitrogen Source on Proline Accumulation, and Drought/Heat Stress

Stress responses in plants are highly dynamic and often involve a complex cross-talk between gene expressions, and a unique reprogramming at the metabolic, and phenotypic levels. It is known that nitrogen source increases proline quantity, however, the mechanistic explanation is mostly imprecise. Water and heat stresses trigger sucrose synthases (*SUS1* and *SUS4*), *glucan, water dikinase 2* (*GWD2*) as well as *P5CS1*. Gurrieri et al. (2020) have shown that wild-type plants and loss-of-function mutants show no differences in transitory starch and cell wall carbohydrates and in the total amino acid content under water deficit conditions. But water-soluble sugars and proline contents decline in mutants in comparison with wild-type plants. Their results indicate that GWD2 strengthens the involvement of SUS1 concerning osmotic stress and higher contribution of soluble sugars than proline in osmotic adjustment under drought stress (Gurrieri et al., 2020). Also, a putative interaction between proline and soluble sugars has been noticed (Gurrieri et al., 2020). However, the nature of the interaction between soluble sugars and proline is yet to be ascertained. Proline concentration in phloem exudates, uptake of nitrogen and absorbed nitrogen from the soil has been determined under water deficit conditions in *Trifolium repens* (Lee et al., 2009). Under drought stress, proline content in phloem exudates has been found enhanced, with a concomitant decrease in nitrate reductase activity in roots. The results prove that proline transport to roots *via* phloem was enhanced due to drought stress conditions which are governing the uptake and assimilation of newly absorbed nitrogen (Lee et al., 2009). However, if such a link exists or not between high day and high night temperature stresses, proline transport to phloem and nitrogen nutrition is largely elusive. Exogenous supply of proline reduces the levels of malonaldehyde and H_2_O_2_ under HTS in tomato, it improves the water use efficiency, fruit quality attributes like acidity and total soluble solids, and final yields (Tonhati et al., 2020). It emerges therefore, that proline alleviates HTS by improving the oxidative stress damage. Lopez-Delacalle et al. (2021) noticed that proline and ascorbate pathways act highly in a synchronous fashion to maintain cellular redox homeostasis under salt and heat combination stresses in tomato. They identified the transcription factor families like the basic leucine zipper domain (bZIP), zinc finger cysteine-2/histidine-2 (C2H2) and trihelix as modulators of the up-regulated genes when salt and heat stresses were given in combination. Rivero et al. (2004) studied the effect of different nitrogen sources (NO_3_^–^ or NH_4_^+^) on heat stress tolerance due to the accumulation of proline in tomato leaves. They noticed that proline has accumulated due to increased *P5CS* and *OAT* expressions with a concomitant decrease in *ProDH* and *P5CDH*. In NH_4_^+^ fed-plants, at 35^°^C, proline and biomass of plants was higher in comparison with NO_3_^–^ fed-plants. Tomato plants fed with NH_4_^+^ as a source of nitrogen unveil higher temperature tolerance (35^°^C) than the plants that were fed with NO_3_^–^ (Rivero et al., 2004). Such a tolerance was correlated to the higher accumulation of proline when NH_4_^+^ was fed to the plants than nitrate (Rivero et al., 2004). The experiments point out that the source of nitrogen is crucial for proline accumulation under high temperature and in imparting tolerance to HTS. In line with this, Dellero et al. (2020) noticed downregulation of three *P5CS1* genes in source leaves with reduced commitment of nitrogen source toward proline biosynthesis in *Brassica napus*. Contrary to this, *ProDH* genes were upregulated by carbon starvation (dark-induced senescence) compared to early senescing leaves. It is evident that besides nitrogen status, dark to light transition and stress response jointly modulate the differential expression of *P5CS* and *ProDH* genes associated with proline synthesis and degradation.

## Genome Editing by CRISPR/Cas9 and High Temperature Stress Tolerance

Besides genetic engineering technologies, editing of one or more genes through multiplex-multigene CRISPR/Cas9 system is an encouraging approach for generating crop plants tolerant to abiotic stresses including HTS. While *P5CS* and *P5CR* are important positive regulators in proline biosynthesis, ProDH is the rate limiting step in its degradation. Both *P5CS* and *P5CR* have not been subjected to editing, but are promising candidate genes. Transcript levels of *OsProDH* were higher in roots and leaf blade, and HTS repressed the expression of *ProDH* in rice (Guo et al., 2020). While overexpression of *OsProDH* (single copy gene in rice) decreased proline quantity, a knockout mutant (created using CRISPR/Cas9) increased the proline content (Guo et al., 2020). CRISPR-edited lines displayed better resistance in comparison with wild-type rice plants. These results imply that *OsProDH* negatively modulates heat stress tolerance in rice seedlings by regulating proline catabolism (Guo et al., 2020). Knockout mutants accumulate less H_2_O_2_ than the overexpressed lines. It appears therefore, that proline accumulation quenches the ROS, thereby imparts thermal tolerance. Paralogous genes must be kept in mind which may get targeted if not protected carefully (Saikia et al., 2020). Care must therefore be exercised while selecting target genes through pre-CRISPR analysis for multiplex-multigene editing while making efforts to manipulate proline metabolism. The gene editing procedures including TALENS/ZFNs or CRISPR/Cas9-based multiplexed editing have shown great promise in deletion of candidate motifs associated with stress tolerant varieties in tomato (Tran et al., 2021). To develop climate resilient heat tolerance lines, undertaking precision genome editing of proline biosynthesis and degradation targets is necessary to attain redox, energy homeostasis and to scavenge ROS. Although Tran et al. (2021) have attempted to understand this in the manipulation of hybrid proline-rich protein 1 (HyPRP1) domains, engineering such domains for coping up with climate changes needs to be further heralded.

## Conclusion

Despite being a proteinogenic amino acid, proline has a wide range of activities in plants, as an osmoprotectant for osmotic adjustment under conditions of salt, drought and temperature stresses. Recent evidence surmises that proline reduces the production of ROS, through quenching singlet oxygen and superoxide radicals in thylakoids. It is widely acknowledged that proving this evidence in whole plants is challenging. As a result, much more research is needed to show that proline is a good quencher matching to the properties of non-enzymatic antioxidant compounds. Proline cycle acts as a shuttle to transport redox couples from mitochondria to cytoplasm and back. Both synthesis and degradation of proline appear to be highly critical during plant growth and stress conditions. The enzymes of the biosynthetic pathway have not been completely characterized under diverse abiotic stress conditions and more studies on protein-protein interactions, membrane transporters are necessary. Enzymes involved in proline catabolism interact with Trx, but other interacting partners need to be uncovered to understand stress response. Recent findings filled the gaps in our understanding of proline cycle and production of ROS and their homeostasis in attaining heat tolerance. Proline catabolism has been implicated in cancer biology in humans and abiotic stress tolerance in plants. Therefore, understanding the biochemical mechanisms of how proline improves the stress tolerance in plants and also human health is vital for us. We need to target to increase proline under short-term stress to quench ROS and to attain proline homeostasis under prolonged stress and then yield stability through a balanced redox potential. But it appears that proline metabolism can only be an adjunctive target for generating heat stress tolerant plants.

## Author Contributions

PBKK and NS conceived the idea and wrote a major part of the review article. PBKK, PS, PR, and NS analyzed the data, interpreted, and wrote the manuscript. NS has generated the heat map. PS has analyzed the bioinformatics data, created the figures, and interpreted the results. All authors have critically analyzed the manuscript, read and approved it.

## Conflict of Interest

The authors declare that the research was conducted in the absence of any commercial or financial relationships that could be construed as a potential conflict of interest.

## Publisher’s Note

All claims expressed in this article are solely those of the authors and do not necessarily represent those of their affiliated organizations, or those of the publisher, the editors and the reviewers. Any product that may be evaluated in this article, or claim that may be made by its manufacturer, is not guaranteed or endorsed by the publisher.
